# Supplementary material for: Does mathematics training lead to better logical thinking and reasoning? A cross-sectional assessment from students to professors
Source: PLoS One. 2020 Jul 29;15(7):e0236153. doi: 10.1371/journal.pone.0236153 (PMC7390332; doi:10.1371/journal.pone.0236153)
Supplement: Attachment — Submitted filename: Response to Reviewers.docx [file pone.0236153.s002.docx]

| **Reviewer comment** | **Author response** | **Changes to manuscript** |
| --- | --- | --- |
| **Reviewer #1** |  |  |
| I think this is a very good and interesting manuscript trying to answer an important research question. I propose some changes that I believe should be applied before publication. |  |  |
| 1. Each reasoning bias is measured with only one problem. In reasoning research, it is rather common to measure each type of reasoning problem with a series of structurally equivalent reasoning problems, so the results will be independent of contexts effects and will be generalizable to that type of problem. Here, the authors only measured each reasoning bias with one single problem and this might be problematic (see, for example: Fiedler & Hertel, 1994). I think this can be addressed by simply discussing it in the limitation section. | This is not an accurate characterisation of the problems used in this study. Three problems (Lily Pads, Widgets Bat and Ball) measured of Cognitive Reflection. Four problems measured probability reasoning (Hospitals, Birth Order a & b, Coin Tosses). The remaining problems (Four Cards Problem, Two Drivers, Petrol Station, Jack looking at Anne) measured problem solving ability, but admittedly different types of problem solving.  When the issue under investigation in this study has been examined in previous research (e.g., Inglis & Simpson, 2005), only one problem (Four Cards) was used. Our use of 11 problems definitely marks an expansion of the theoretical base of the possible effects of mathematics training. Certainly we attempted to broaden the applicability of the results beyond one reasoning bias.  Given the nature of our data collection method (all student data was collected in the context of a class), we were limited in the amount of time that could be devoted to this task. Our aim was to limit the time required of each participant to less than one hour. Any attempt to expand the number of problems per reasoning bias would require additional testing time, and so could require a different mode of data collection. Nonetheless, we have considered this possible limitation of the results in the Limitations section. | This issue is discussed in the Limitations section (lines 869-875). |
| 2. This is rather a minor issue, but the discussion on the CRT problems is not up-to-date (page 7). Most recent experiments on dual process theory suggest that people who are able to correctly solve these reasoning problems (including the CRT) do so intuitively, and not because they engaged in careful deliberation (Bago & De Neys, 2019). Intelligence made people have better intuitive responses (Thompson, Pennycook, Trippas & Evans, 2018). Similarly, this problems persists in the discussion about reaction times (page 25). Longer reaction times does not necessarily mean that people engaged in deliberation (see: Evans, Kyle, Dillon & Rand, 2015). Response time might be driven by decision conflict or response rationalization. These issues could be clarified with some changes in the wording or some footnotes on page 7 and 25. Furthermore, it would be interesting to have a discussion on how mathematical education helps people overcome their biases. Is it because it creates better intuition, or helps people engage in deliberation? An interesting question this manuscript does not discuss. It’s on the authors whether or not they discuss this latter point now, but the changes on page 7 and 25 should be made. | Fair points. | Wording on page 7 (lines 154-155) and page 25 (lines 614-619) has been altered to reflect these points.  As a result of inserting 3 additional references to address this issue, the references in the paper required re-numbering (see lines 907-1005). |
| 3. A more serious problem is the rather small sample size (especially in the more advanced groups). This small sample size makes the appearance of both false negatives and false positives more likely. Perhaps, the authors could compute the Bayes Factors for the chi-square or logistic regression test, so we can actually see how strong the evidence is for or against the null. This is especially important as the authors run a great number of explorative analysis (Table 3), and some of those results might need to be interpreted with great caution (depending on the Bayes Factor). | This is as big a sample size as was possible in the one university we had access to.  Nonetheless, we have conducted additional Bayes-equivalent analyses. In all cases these additional analyses supported the conclusions reached on the basis of the chi square analyses. Thus there was no cause for reinterpreting the results, nor any need for additional caution in interpretation. | Bayes Factor values are reported alongside the chi square results (see lines 408, 420-421, 452, 459, 462, 485, 492-493, 512, 522, 531, 554). |
| The graphs are not looking good, they should comply with APA formatting. At the very least, the axis titles should be meaningful and measure units should be written there. | Agree | Both Figures have been re-drawn. |
| The presentation order of the problems is quite unusual; why isn’t it random? Why did the authors decide on this order? | “Unusual” is a bit vague. If presentation order is an issue it could be dealt with in a future study. This point addresses a different issue to the one in focus here. | No change to manuscript |
| **Reviewer #2** |  |  |
| The study reported in this paper compared five groups of participants with varying levels of mathematical expertise on a set of reasoning tasks. The study is interesting and informative. It extends the current literature on this topic (which is reviewed very nicely in the introduction). However, there are some issues with the current analysis and interpretation that should be resolved prior to publication. I have therefore recommended major revisions. My comments are organised in the order in which they came up in the paper and they explain my responses to the questions above. |  |  |
| 1. Line 114 – “general population” a bit misleading – they were also students but from other disciplines. | Agreed | This phrase has been deleted (line 115) |
| 2. Line 124 onwards reads: “The ultimate question to consider here is: are any skills associated with mathematics training innate or do they arise from skills transfer? Though to investigate how mathematical training affects reasoning skills, randomised sampling and randomised intervention to reveal causal relationships are clearly not viable. With so many possible confounding variables and logistical issues, it is even questionable what conclusions such studies might provide. Furthermore, a firm baseline from which to propose more substantive investigations is still missing.”  I find this paragraph slightly problematic because the current study doesn’t inform us on this ultimate question, so it makes the outline of the current study in the following paragraph feel unsatisfactory. I think the current study is important but prefacing it with this paragraph underplays that importance. And I think a randomised controlled study, although not viable, would give the answers we need because the random allocation to groups would allow us to rule out any confounding variables. Finally, the last sentence in this paragraph is unclear to me. | Agreed | This paragraph has been deleted (line 124) |
| 3. In the descriptions of the five participants groups the authors refer to the group’s level of interest in mathematics, but this seems like an overgeneralisation to me. Surely the introductory group could contain a biology student who also happens to be good at mathematics and very much enjoy it? I would be more comfortable with the descriptions if the parts about interest level were removed. | We largely agree with the reviewer on this point, so we have deleted reference to interest level, but only in the Introductory and Standard groups. We argue that interest is still relevant for the two Advanced groups because students only take these classes if they have a specific interest in mathematics. | Relevant changes are in lines 333 and 337. |
| 4. How many of the 123 first year students were in each of the three first year groups? | Information (n) is in Table 1. | No change to manuscript |
| 5. Line 313 – the standard group is referred to as “university mathematics students”, but they are not taking mathematics degreed. | Agreed | Relevant changes are in lines 337 and 338 |
| 6. Line 331 - what is a practice class? | Clarification provided. | Clarification provided in “Participants” section (lines 357-363). |
| 7. Were the data collection settings quiet? From the description it sounds like groups of participants were completing the study at the same time in the same room, but the authors should make this explicit for the sake of the method being reproducible. E.g. how many students were in the room at the time? | Further details have been provided to address this point. | Clarification provided in “Participants” section (lines 354-364). |
| 8. Line 355-356 – the authors should not use the term “marginally worse” because this is statistically inappropriate – in a frequentist approach results are either significant or non-significant. | Agreed | This phrase has been removed (line 391). |
| 9. Line 340 – “approximate completion times were noted.”  This doesn’t sound rigorous enough to justify analysing them. Their analysis is interesting, but the authors should remind readers clearly whenever the response times are analysed or discussed that their recording was only manual and approximate. | Agreed | Language has been modified to suit. See “Procedure” (lines 374-376), “Results” (lines 400, 402, 405) and first paragraph of “Discussion” (lines 604, 608-610). |
| 10. I suggest replacing Figure 1 with a bar chart showing standard error of the mean on the error bars. A table with mean score out of 11 and the standard deviation for each group may also be useful. Figure 2 should be a scatterplot rather than a box and whisker plot. | We agree with the suggestion regarding Figure 1. We disagree with the suggestion about Figure 2. A scatterplot would imply that both variables are continuous. Certainly response time is continuous, however, it is less clear that the group variable is continuous. Although we would argue that the group variable is roughly correlated with amount and complexity of mathematical experience, the differences between the particular points on this continuum are not equal, and so this would make interpretation of a scatterplot of the time x group data problematic. | Both figures have been re-drawn. |
| 11. Was the 0-11 total correct score approximately normally distributed across the full sample? | Yes | No change made to manuscript. |
| 12. Chi square analysis requires at least 5 cases in each cell, was this met? It seems not since Table 1 shows lots of cells in the “no response” row having 0% of cases. | The requirement of Chi square is not that the number of observed values in a cell is 5 or more, but that the number of expected values in a cell is 5 or more. This requirement was satisfied in most cases. Nonetheless, to minimise the number of cells that did not meet this requirement, the error response categories (Standard Error, Other Error, Blank) were collapsed into one category. This did not affect any of the analysis outcomes. | A comment to this effect is included in the Results section (lines 410-416). |
| 13. The chi-square analyses should be followed up with post hoc tests to see exactly where the differences between groups are. The descriptions as they stand aren’t that informative (as readers can just look at Table 1) without being backed up by post hoc tests. | Agreed, however this is only relevant to three problems (Four Cards, Two Drivers and Jack looking at Anne) where comparisons between specific groups were originally considered. Post hoc tests are now reported for these comparisons. | Post hoc chi square results are reported in lines 435-436, 522 and 554-555. |
| 14. For each chi square analysis in the text, I would find it easier to read if the test statistics came at the top of the paragraph, before the description. | Where relevant, this has been actioned. Where this has not been changed, the chi square information is presented with the description of the overall result for a particular problem, and this was already presented first. | Changes to the position of the chi square results are in lines 419-421. |
| 15. Line 381-383 – “Of note, also, is the relatively low proportion of those in the higher training groups who, when they made an error, did not make the standard error, a similar result to the one reported by Inglis and Simpson [11]."  I think this is supposed to say that a low proportion did make the standard error or that a high proportion did not make the standard error. | Agreed | Relevant wording amended (line 436) |
| 16. Line 403 - p values this small should be reported as p < .001 rather than p = .000 since they aren’t actually 0. | Agreed | This change has been made (line 461) |
| 17. Line 476 – “…if a particular outcome variable was predicted significantly by a particular predictor variable, the converse relationship was also observed”  Isn’t that necessarily the case with regression analyses, like with correlations? | Agreed | This sentence has been deleted (line 566). |
| 18. I don’t think the logistic regression analyses add much to the paper and at the moment they come across as potential p-hacking since they don’t clearly relate to the research question. To me they make the paper feel less focused. Having said that, there is some interesting discussion of them in the Discussion section. I’d recommend adding some justification to the introduction for why it is interesting to look at the relationships among tasks (without pretending to have made any specific hypotheses about the relationships, of course). | We agree with this comment. | Two sentences have been included at the end of the Introduction to indicate our intention to explore this issue (lines 314-321). |
| 19. Line 509 would be clearer if it read “between these groups and the introductory and standard groups” | Agreed | Wording amended (line 607) |
| 20. Lines 597 – 620 - This is an interesting discussion, especially the suggestion that advanced calculus may be responsible for the development. No development in reasoning skills from the beginning of a mathematics degree onwards was also found by Inglis and Simpson (2009), who suggested that the initial difference between mathematics and non-mathematics undergraduates could have been due to pre-university study of mathematics. Attridge & Inglis (2013) found evidence that this was the case (they found no difference between mathematics and non-mathematics students at age 16 but a significant difference at the end of the academic year, where the mathematics students had improved and the non-mathematics students had not).  Could the authors add some discussion of whether something similar may have been the case with their Australian sample? E.g. do students in Australia choose whether, or to what extent, to study mathematics towards the end of high school? If not, the description of the groups suggests that there were at least differences in high school mathematics attainment between groups 1-3, even if they studied the same mathematics curriculum. Do the authors think that this difference in attainment could have led to the differences between groups in the current study? | Agreed | Discussion of this point added (lines 702-790). |
| 21. Line 617 – “Intensive training has been shown to impact the brain and cognition across a number of domains from music, to video gaming, to Braille reading [31].”  Reference 31 appears to only relate to music. Please add references for video gaming and Braille reading. | References added | See lines 805-806 |
| 22. I recommend editing the figures from SPSS’s default style or re-making them in Excel or DataGraph to look more attractive. | This is a fair point. | Both figures have been re-drawn using Origin. |
| 23. I cannot find the associated datafile anywhere in the submission. Apologies if this is my mistake. | We had assumed that the information provided in Table 1 was sufficient for most purposes. We can see now that the raw frequencies might also be valuable, and so have prepared a supplementary data file the includes these. | See supplementary data file (“raw daw.xlsx”). |
