# Supplementary material for: Does mathematics training lead to better logical thinking and reasoning? A cross-sectional assessment from students to professors
Source: PLoS One. 2020 Jul 29;15(7):e0236153. doi: 10.1371/journal.pone.0236153 (PMC7390332; doi:10.1371/journal.pone.0236153)
Supplement: Attachment — Submitted filename: Response to Reviewers.docx [file pone.0236153.s003.docx]

| **Reviewer comment** | **Author response** | **Changes to manuscript** |
| --- | --- | --- |
| **Reviewer #2** |  | **Please note that line numbers refer to the “Manuscript” document. For some reason the “Revised Manuscript with Track Changes” document is showing some different line numbers.** |
| 1. On lines 96, 97, 100 and 102, the references to “general population” should be changed to reflect the fact that these participants were non-mathematics (arts) students. | OK | Recommended changes to phrasing have been made (lines 96, 97-98, 100, 102-103) |
| 2. Line 306 – change “mathematics students” to “university students”. | OK | Recommended change made (line 307) |
| 3. The method section doesn’t specify the gender split and mean age of the sample. | The ethics committee that gave approval for this study recommended against recording gender unless it was pertinent to the issue under examination. We decided it was not pertinent, so gender was not recorded. Age was recorded, and is now included. | Mean age of the group is now included in Table 1. |
| 4. Table 3 - values the p values listed as .000 should be changed to <.001. | OK | Recommended changes have been made to Table 3. |
| 5. Table 3 - I suggest repeating the list of problem numbers and names in the legend. It may make for a long legend but would make it much easier for the reader to interpret the table. | OK | This information has now been included as a note in Table 3 (lines 505-507) |
| 6. I am not sure what the new post hoc tests are comparing. What I expected was to see group 1 compared to groups 2, 3, 4 and 5, and so on. This would tell us which groups are statistically different from each other. At the moment we only know from the overall chi square tests whether there are any differences among the groups or not, we don’t know specifically which groups are statistically different from each other and which ones are not. We only have the authors’ interpretations based on the observed counts. | OK | The requested post hoc information is now included in Table 1, described in a note to the table (lines 401-405, and referred to in lines 382-383. |
| 7. Line 584 - change “performance was correlated with training” to “performance was related to training” to avoid any confusion since a correlation analysis was not performed. | OK | The requested change has been made (line 592). |
| 8. Data file – I had expected the data file to give the raw data rather than summary data, i.e. with each participant in a separate row, and a column indicating their group membership, a column giving their age, a column for sex etc (including all the demographics mentioned in the method), and a column for each reasoning question. This would allow other researchers to replicate the regression analyses and look at other relationships within the dataset. Without being able to replicate all analyses in the paper, the data file does not meet the minimal data set definition for publication in PLOS journals: hYps://journals.plos.org/plosone/s/data-availability. | OK | The requested form of the raw data file is now included as supplementary materials. |
